# Rectal Neuroendocrine Tumours: A 10-Year Review of Clinical Presentation, Pathological Features, and Treatment Outcomes from a Tertiary Care Cancer Centre in Western India

**DOI:** 10.1007/s13193-025-02323-7

**Published:** 2025-05-20

**Authors:** Katyayani Kumari, Vivekanand Sharma, Ashwin DeSouza, Mufaddal Kazi, Ankit Sharma, Vikram Anil Chaudhari, Munita Bal, Avanish Saklani

**Affiliations:** 1 Department of Surgical Oncology, Tata Memorial Hospital, Mumbai, India; 2 Department of Surgical Oncology, Breach Candy Hospital, Mumbai, India; 3 Department of Pathology, Tata Memorial Hospital, Mumbai, India

**Keywords:** Neuroendocrine tumours, Rectal cancer, Multimodality treatment

## Abstract

Rectal neuroendocrine tumours are rare but increasing worldwide. However, there is limited data from regions like the Indian subcontinent, where clinical presentations and outcomes may differ due to unique demographic and biological factors. This study aimed to characterize rectal neuroendocrine tumours in our region, focusing on clinic-pathological presentation, and treatment outcomes. This was an observational single-centre retrospective cohort study from a high-volume tertiary care centre in Western India. Sixty-five consecutive patients with rectal neuroendocrine tumours treated between 2013 and 2023 were included. The main outcome measures were overall survival and disease-free survival. Secondarily, we tried to evaluate the impact of pathological grade and surgery-type on survival outcomes. The median age at diagnosis was 50 years, younger than the global median (56–57 years), with a male predominance (80%). Majority of patients were symptomatic and had locally advanced disease, with 64% showing metastatic spread. Pathologically, 82% of tumours were classified as Grade II/III, with a high median tumour size (3.7 cm) and elevated serum Chromogranin A levels. Multimodal treatment, including surgery and adjuvant therapies, was utilized for most patients. Of the 41.5% who underwent surgery, 70% had sphincter-preserving procedures. The median overall survival for the entire cohort was not reached, but 3-year and 5-year overall survival rates were 91% and 85%, respectively. Grade III tumours had significantly poorer outcomes, with a 5-year survival of 57% compared to nearly 100% in Grade I and II tumours. Apart from its retrospective nature, our study may have limited generalizability due to potential referral bias, and the lack of detailed pathological subclassification would be an opportunity for future research. As the first study from the Indian subcontinent we highlight how our patients presented at a younger age with advanced, aggressive disease. Multimodal approach could improve outcomes even in advanced disease.

## Introduction

Neuroendocrine tumours (NETs) are a group of tumours that arise from the neuro-endocrine cells and show a wide range of pathological heterogeneity, varied aggressiveness of biological behaviour and propensity for being malignant. Owing to their neuroendocrine origin, these often exhibit a wide range of clinical features, reflecting both neural and endocrine components. NETs of the rectum comprise nearly 12–25% of all gastrointestinal NETs and 2% of all colorectal cancers [1]. Although uncommon, their incidence appears to be on the rise worldwide, as noted in reports from the USA [2], Australia [3], Europe [4, 5], and Asia [6]. Globally, the majority of them are identified incidentally in asymptomatic patients, as seen in nearly 0.05–0.17% of screening colonoscopy participants in the UK [7] and Poland [8].

Due to their rarity, prospective evidence is limited and most treatment decisions are guided by retrospective studies and consensus guidelines. Much of the published data though comes from countries with population-based colonoscopy programs, with little published evidence from large geographies like Africa and the Indian subcontinent, which are home to a significant proportion of the world’s population [9]. Since reports have identified that there is a racial predisposition of people from African [10] and Asian origin [9] to develop NETs, establishing the disease burden in these areas is essential to address this knowledge gap and, key to ensure representative data is used for guiding clinical decision making.

The current study is a 10-year audit of rectal NETs treated at a high-volume cancer centre in Western India. We aimed to identify the clinical presentation patterns, pathology of disease, and outcome of the patients in this geographical region.

## Materials and Methods

### Study Design, Settings, and Participants

This was a single-centre retrospective cohort study from a tertiary care referral colorectal cancer hospital in Western India. We screened a prospectively maintained neuroendocrine database and included all consecutive patients who were diagnosed to be suffering from pathologically confirmed rectal NET and took cancer directed treatment (medical and surgical) with us between January 2013 and December 2023. Adeno-carcinomatous and mixed-adeno and neuroendocrine histologies were excluded.

### Management

All patients with suspected colorectal cancer underwent clinical examination, serum carcinoembryonic antigen (CEA) levels, and endoluminal evaluation with colonoscopy. Pathological evaluation and immunohistochemistry using Chromogranin, Synaptophysin, and INSM1 staining (including review of slides or blocks made elsewhere) were done by specialist colorectal onco-pathologists. After confirming the neuroendocrine origin, further characterization was performed, including WHO grading, MIB index/Ki-67 estimation, and serum chromogranin A (CgA) level measurement. Staging scans (for local and distant disease), and when applicable, nuclear medical imaging were requested (based on grade and MIB index) as per recommendations of the multidisciplinary team (MDT). At our centre, for low-grade disease, a DOTA PET CT scan (Gallium-68 DOTATATE scan) is usually offered. For Grade II disease, we combine this with 18-FDG-PET-CT, and for Grade III disease, only a 18-FDG-PET-CT is recommended.

After MDT review of the patient symptomatology, performance status, grade of disease, and metastatic burden, treatment was recommended. Apart from the neuroendocrine and colorectal MDT, plans were also discussed with the hepatobiliary MDT in case of suspected liver involvement. At our centre, for all lesions, surgery is offered for functional or symptomatic lesions if R-0 resection is possible without undue morbidity. In small, non-functional and asymptomatic Grade I lesion, observation is recommended if patient is compliant with follow-up. In advanced Grade I/II, long-acting somatostatin analogues are recommended for symptom control. Additionally, for Grade II lesions, peptide receptor radionucleotide therapy (PRRT) is used for symptom control and disease downstaging. In Grade III lesions or in high-burden metastatic disease, neoadjuvant chemotherapy or neo-adjuvant chemoradiotherapy (in low rectal lesions) is usually employed as is done for adenocarcinomas. Post neo-adjuvant treatment, if disease becomes amenable to R-0 resection or the patients has severe symptoms uncontrolled by best medical management, surgery is suggested.

Hospital electronic records of in-person visits or teleconsultations were primarily used for documenting course of treatment, recurrences, and deaths. Telephonic contact was made when patients did not attend their scheduled visits.

### Variables and Outcomes

The demographic parameters recorded were age, sex, clinical presentation, serum tumour markers (serum CEA and CgA level), and performance status on the Eastern Cooperative Oncology Group scale. We noted tumour size, morphology, World Health Organisation (WHO) grade and proliferation indices, and tumour location. The AJCC staging and treatment characteristics, including the type of surgical and non-surgical management (chemotherapy, or chemo-radiation, or PRRT), were also documented. Median follow-up was calculated using reverse Kaplan–Meier method.

The main outcome measures were overall survival (OS) and disease-free survival (DFS). Local recurrence was defined as tumour recurrence within the pelvis (or in the liver, in case of disease arising in the bed of previous liver metastatectomy), while extra-pelvic or non-regional nodal relapses or discontinuous new liver lesions were labelled as distant failures. Disease-free survival (DFS) was calculated from the date of first treatment to the date of recurrence, death or the last follow-up date, and overall survival (OS) from the date of first treatment to the date of death or date of last follow-up. Patients were censored at their last follow-up.

Secondarily, we tried to evaluate the impact of grade of disease on survival and type of surgery on the patient’s disease-free and overall survival.

### Statistics

Data were recorded and analysed using the Statistical Program for Social Sciences®, version 26 (IBM Corporation, Armonk, NY, USA). Numerical variables are presented using median and interquartile range and comparisons made using the Kruskal–Wallis test. Categorical data are described using numbers and proportions, and comparisons made by the chi-square test. Time-to-event variables are summarized by Kaplan–Meier curves and compared using the log-rank test. A *p*-value ≤ 0.05 is considered statistically significant.

### Ethics

The study protocol was in accordance with the ethical standards of institutional research and the 1964 Helsinki Declaration and its later amendments. Each patient signed a written informed consent during treatment and surgery. The study followed the STROBE (‘Strengthening the Reporting of Observational Studies’) guidelines for observational studies [11].

## Results

During the study period, a total of 65 patients took treatment at our centre for rectal NET. The median age of the participants was 50 years (interquartile range 28–67), and 80% of the participants were males.

### Baseline Characteristics

Most patients presented with symptoms like rectal bleeding (65%), pain (46%), and altered bowel habits (41%). Morphologically, 66% patients had low rectal disease, with nearly half of the cohort presenting with an ulcero-proliferative growth. The median tumour size was 3.7 cm (range 0.25–14 cm). The growth was limited to the lateral wall in a third of the patients, but was annular in 20% of the patients. Median serum CgA level was elevated at 63.6 ng/mL (normal < 39 ng/mL). Microscopically, nearly 80% patients presented with Grade II (46%) and Grade III disease (35%). The median MIB index was 10% (range 1–95, IQR 4.5–27.5), indicating a high proliferative rate. The baselines characteristics are also defined in detail in Table 1.

**Table 1:** Baseline characteristics of the patients

Characteristic	*N* = 65
Age (years); median (range)	50 (28–67)
Gender	
Male	52(80%)
Female	13(20%)
Presentation	
Rectal bleeding	42 (64.6%)
Pain while defecation	30 (46.2%)
Altered bowel habits	27 (41.5%)
Loss of appetite	9 (13.6%)
Significant weight loss	11 (16.9%)
Location	
Upper rectum	5 (7.7%)
Middle rectum	17 (26.2%)
Lower rectum	43 (66.2%)
Tumour size (cm); median (range)	3.7 (0.25–14)
Tumour morphology	
Ulcero-proliferative	34 (52.4%)
Polypoidal	28 (43.1%)
Tumour grade	
Grade I	12 (18.5%)
Grade II	30 (46.2%)
Grade III	23 (35.3%)
Tumour location	
Anterior	15 (23.1%)
Lateral	22 (33.8%)
Posterior	14 (21.5%)
Circumferential/annular	14 (21.5%)
Serum CEA (median, ng/mL) (IQR)	2 (1.2–4.7 ng/mL)
Serum Chromogranin A (median, ng/mL) (IQR)	63.6 (36.87–84.9 ng/mL)
Serum CA 19–9 (median, ng/mL) (IQR)	9.59 (2.91–14.31 ng/mL)

### Treatment Outcomes (Table 2)

**Table 2:** Treatment and pathological characteristics

Variable	*N* = 65
Management	
Neo-adjuvant Chemotherapy	16 (24.6%)
Neo-adjuvant Peptide receptor radionucleottherapy	10(15.4%)
Neo-adjuvant Chemo-radiation	7 (10.8%)
Neo-adjuvant long-acting Somatostatin analogues	4 (6.2%)
Combination	4 (6.2%)
Observation	2 (3.1%)
Missing data	3 (4.6%)
**Surgery**	27 (41.5%)
*Upfront*	19 (29.2%)
*Post-Neo-adjuvant chemotherapy*	3 (4.6%)
*Post-Neo-adjuvant chemo-radiation*	3 (4.6%)
*Post-Somatostatin analogues*	1 (1.5%)
*Post-Peptide receptor radionucleotide therapy*	1(1.5%)
**Surgery**	
Transanal excision	3
Anterior Resection	2
Low Anterior resection	12
Intersphincteric resection	5
Abdominoperineal resection	3
Posterior Exenteration	2
Pelvic lymph node dissection done	5
Liver Resection	
Non-anatomical resection	2
Hemi- hepatectomy (Right/left)	2(1/1)
**Pathology (27 operated patients)**	
T stage	
pT1	5
pT2	12
pT3	8
pT4	2
Lymph node stage	
pNx (3 Transanal excision + 1 Low anterior resection operated elsewhere)	4
pN0	4
pN1	19
Lymph node yield (Total/Positive) (median)	17/2
** Adjuvant treatment**	12
Trans-Arterial Chemo-Embolisation	3
Radiofrequency ablation	1
Long acting somatostatin analogue	1
Chemotherapy	7
**Recurrence (n/% of operated patients)**	8 (29.62%)
Local	1 (2.7%)
Distant	7 (25.92%)
Time to recurrence (median)	13.8 months (6.15- 40.24 months)

**Fig. 1:**
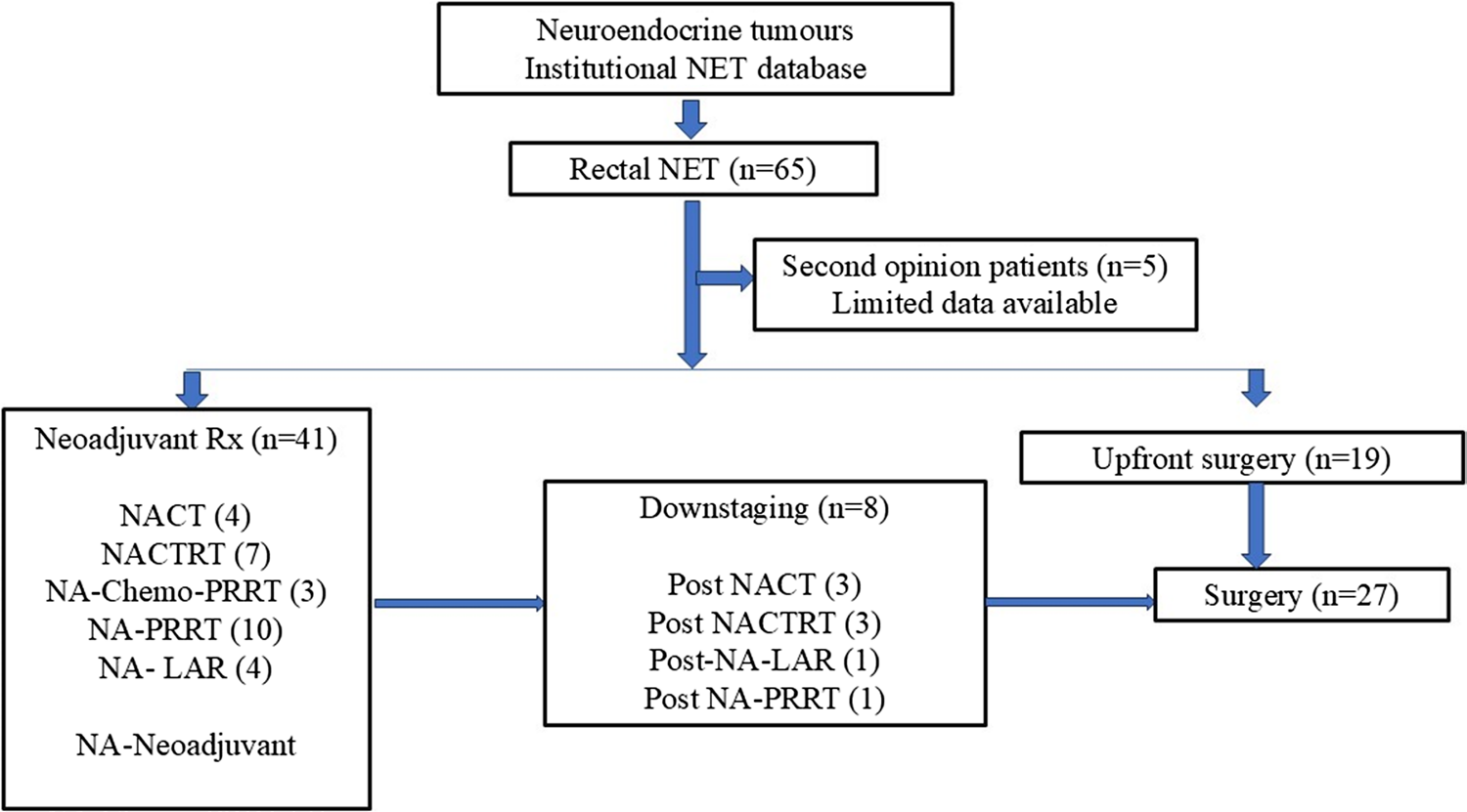
Treatment flow diagram

Most of the patients harboured advanced stage with 64% showing metastatic disease at presentation. Two-thirds of patients (*n* = 41) initially required non-surgical approaches for symptom and disease control (Fig. 1). These included chemotherapy (16 or 40%) (median three cycles) or chemo-radiation (7 or 17%) (same dose as for adenocarcinoma rectum) or PRRT (10 or 25%) (median four cycles) or somatostatin analogues (4 or 10%) (median seven cycles) or a combination of the above. Nearly 20% of these patients (8/41) showed evidence of downstaging and were eligible for surgery subsequently.

Overall, 41.5% (27/65) of patients underwent surgery, with the majority (19/27 or 70%) undergoing upfront surgery. Seventy percent (19/27) of the patients had sphincter saving procedures. Five patients needed an abdominoperineal resection (three as part of posterior exenteration), and an equivalent number required systematic pelvic lymph nodal clearance. Four patients underwent liver metastatectomy. A very small proportion (7.5%) of patients were eligible for organ-sparing treatment, which included either observation (2%) or trans anal excision (4.5%).

On pathological evaluation, 81% (22/27) of the surgically treated patients had disease beyond the muscularis propria, and 70% (19/27) had positive nodal metastases. A median of 17 nodes were isolated in the operated cohort (range 4–41, IQR 10–22), and in them, a median of 2.47 nodes were found to be metastatic. Based on post-operative recovery, performance status, and pathology, nearly 44% of patients received adjuvant treatment, which ranged from systemic chemotherapy, liver-directed treatment (trans-arterial chemoembolization (TACE) or radio-frequency ablation (RFA)), PRRT, or somatostatin analogues. Etoposide–cisplatin and Capecitabine–temozolomide combinations were the most commonly used chemotherapeutic regimens and were used in high grade and node positive disease.

### Survival Outcomes

#### OS—Cumulative and Changes with Grade (Table 3)

**Table 3:** Survival outcomes as per tumour grade

	No	Median size (cm)	Mets	Neo-adjuvant	Surgery (M1)	Curative resection	DFS		OS	
							3 year %	5 year %	3 year %	5 year %
Gd I	12	1.7	5	1	5 (1)	5	100	100	100	100
Gd II	30	3	20	3	15 (6)	15	75	64	100	100
Gd III	23	5	16	4	7 (1)	6	43	43	71	57

**Fig. 2:**
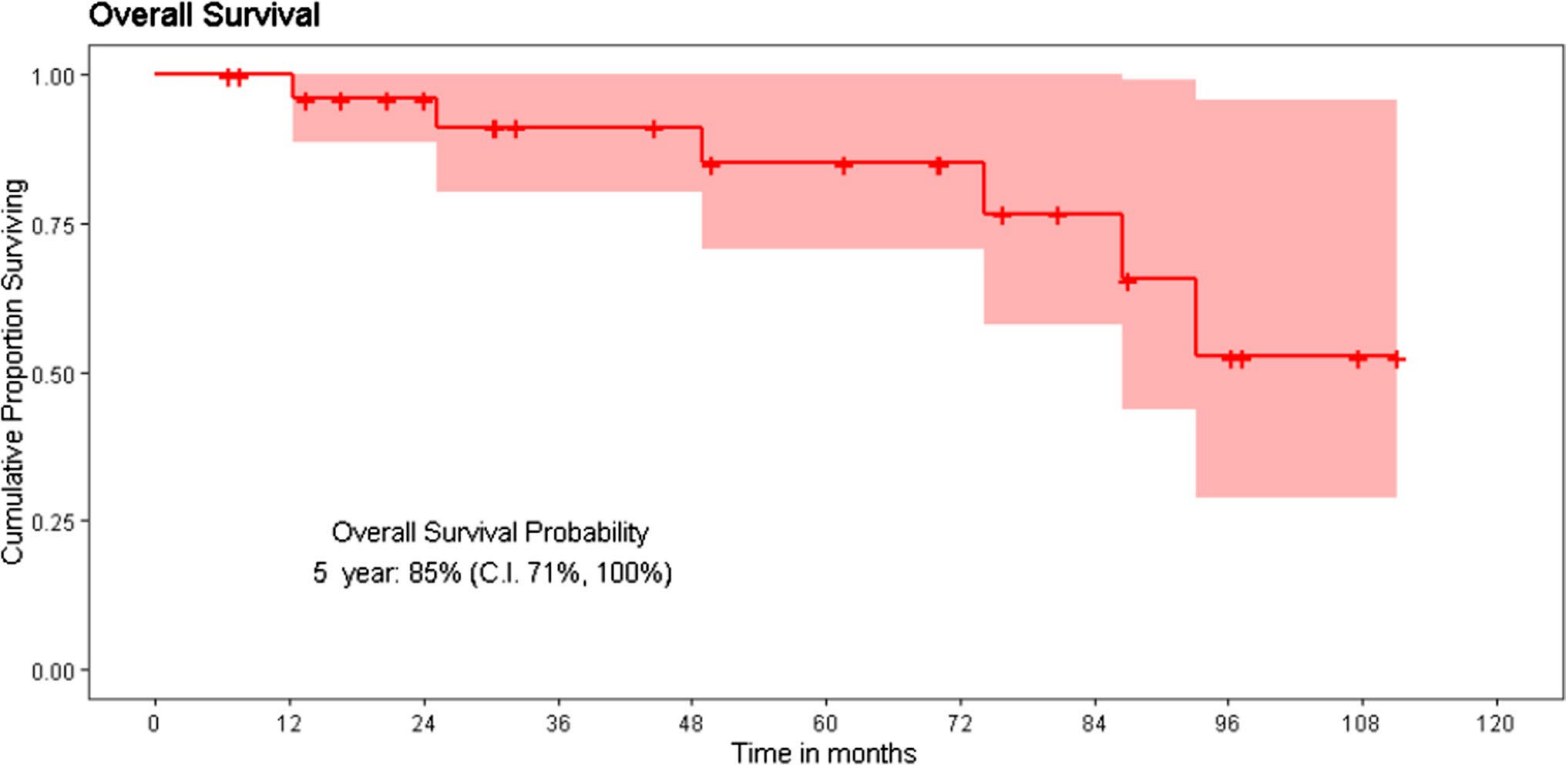
Overall survival of the operated cohort

**Fig. 3:**
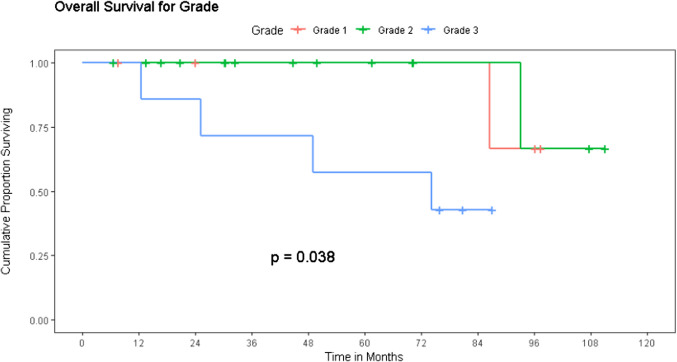
Impact of grade of disease on overall survival

Using the reverse Kaplan–Meier method, the patients had a median follow-up of 70.1 months (95% CI 38.9 months to 101.2 months) using a cut-off date of October 2023. The median overall survival of the entire cohort was not reached and 91% estimated 3-year survival and 85% estimated 5-year survival probability (Fig. 2). The mean survival was 72 months for Grade I disease, 85 months for Grade II disease, and 37 months for Grade III disease. The median overall survival was not reached for Grade I and II disease and was 74.2 months for Grade III disease (Fig. 3). Both Grade I and II disease had a nearly 100% 5-year survival probability. For grade III disease, the 3-year OS probability was significantly poorer at 71%, and 5-year OS probability was reduced to 57% (*p* = 0.04).

#### DFS—Cumulative and Changes with Grade

The mean disease-free survival for the entire cohort was 73.5 months, though the median survival could not be reached. The 3-year and 5-year disease-free survival was estimated to be 70% and 65% respectively.

**Fig. 4:**
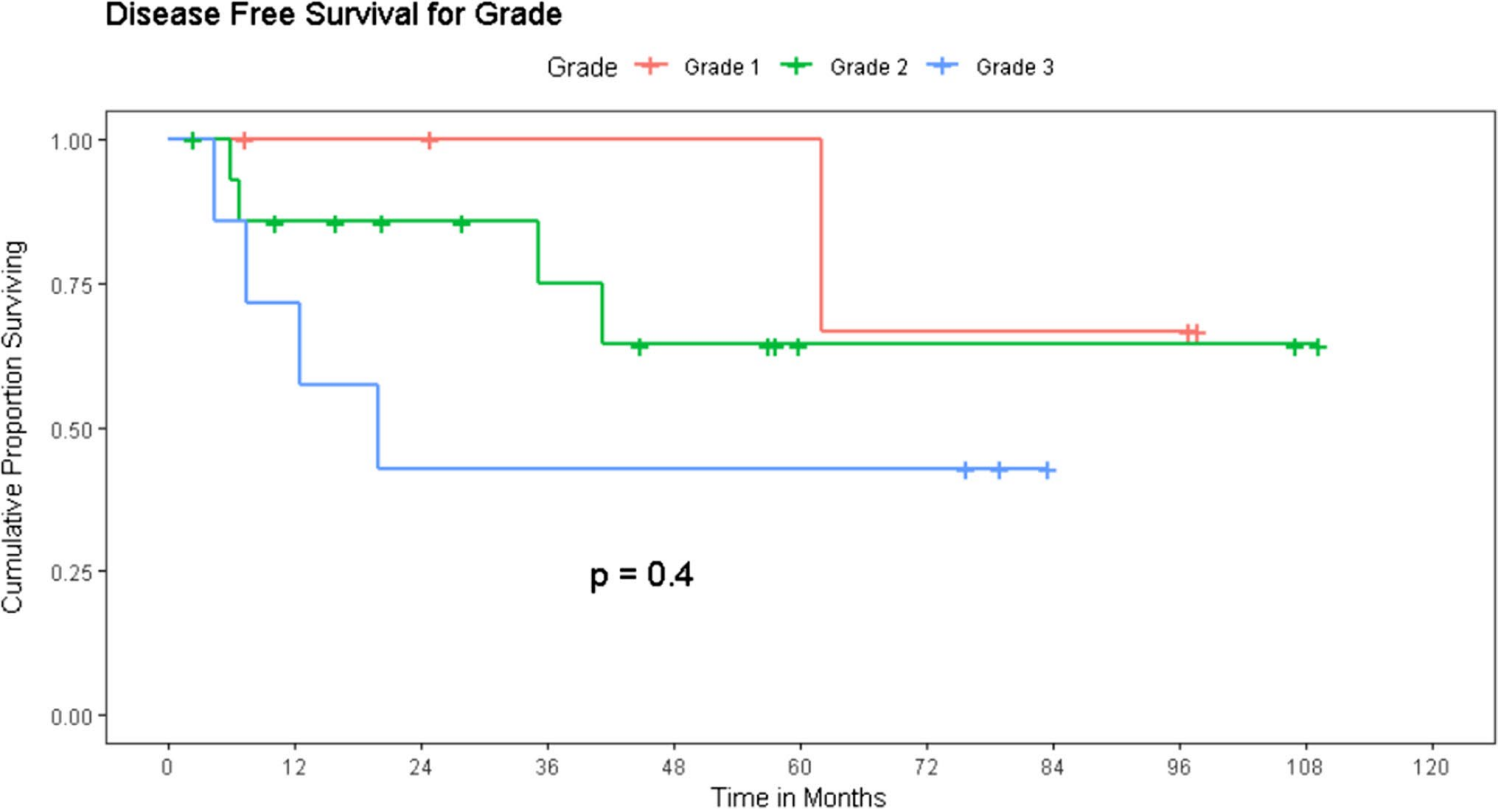
Impact of grade of disease on disease-free survival

The DFS decreased proportionate to advancing grade (Fig. 4). The 3-year DFS probability was 100%, 75%, and 43% for Grades I, II, and III respectively. The 5-year DFS probability was 100%, 64%, and 43% in the same categories, but this reduction did not achieve statistical significance (*p* = 0.4).

#### Impact of Treatment of Survival (Table 4)

**Table 4:** Surgical approaches and outcomes

	M1 Status	CRM + ve	Adjuvant treatment	Recurrence	Local recurrence	DFS (median)		OS	
						3 year %	5 year %	3 year %	5 year %
Sphincter preserving surgery ([LAR/ISR/AR) (*n* = 19)	6	0	8	8	1	74	66	94	94
Extended resection (PE/APR) (*n* = 5)	1	1	4	3	1	40	40	80	60

**Fig. 5:**
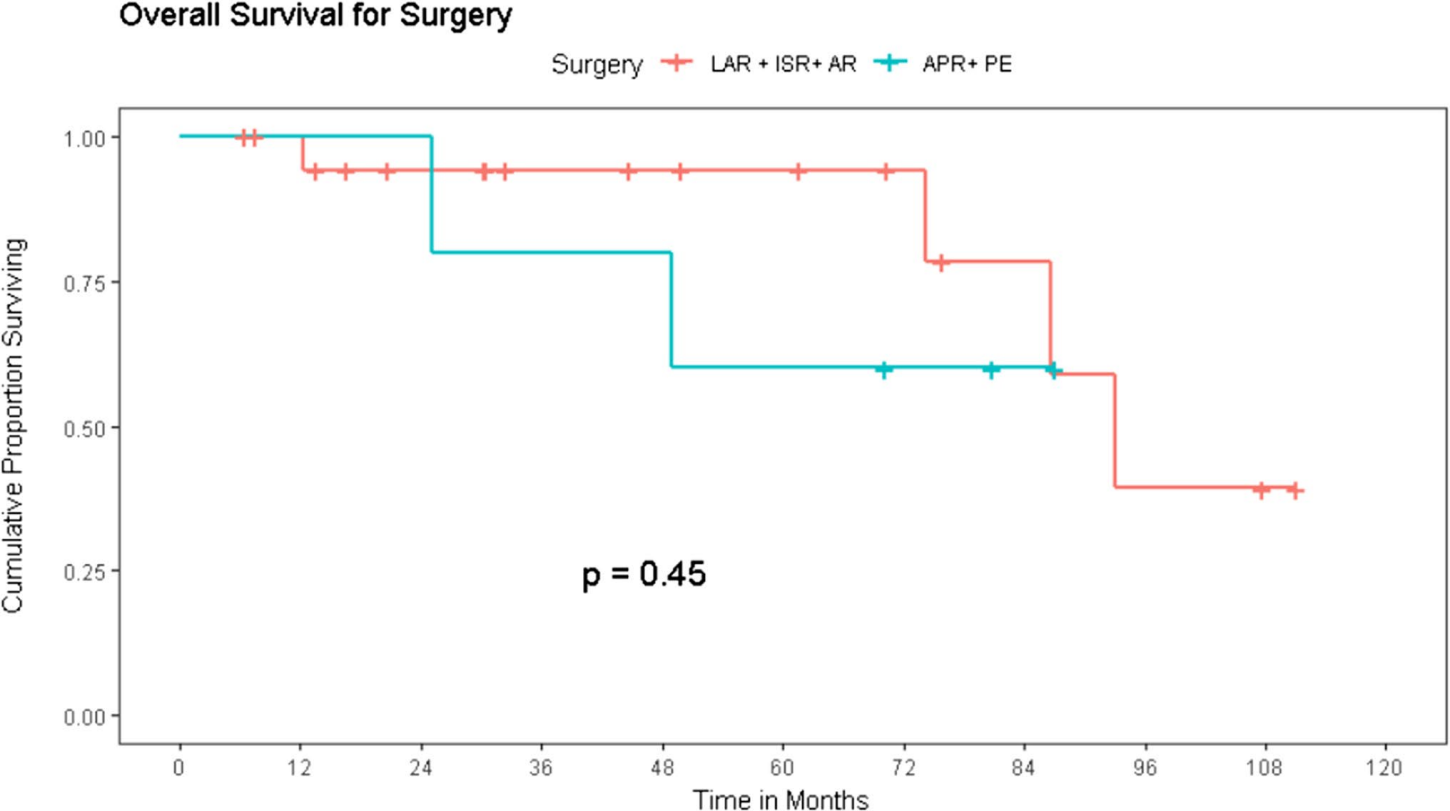
Impact of treatment on survival

Patients with early disease who had local excision had the cohort’s best disease-free survival of 96 months. As local disease worsened, sphincter saving rectal resections had a DFS of 62 months with 3-year and 5-year OS/DFS probability of 94%/74% and 94%/66% respectively. The presence of advanced local disease needing sphincter sacrificing surgeries led to a poorer survival of 20 months DFS with 3-year and 5-year OS/DFS probability of 80%/40% and 60%/40% respectively. While these figures are comparatively poorer, it is noteworthy that in selected patients, aggressive local surgery (APR or exenteration) offered a chance of longer-term survival of 40% at 5 years (Fig. 5).

Median overall survival in the five patients who had pelvic lymph nodal dissection (PLND) was 48 months. It must be recognized however that the numbers in some of these subsets are too small to make any meaningful assumption. Eight patients experienced recurrences. Most common site of recurrence was distant (six) with one experiencing local and one both concurrent local and distant recurrence. Most common sites of distant recurrence were liver (five), bone (four), and two of nodal, peritoneal, and pancreatic metastases. Six patients received salvage treatment, three surgically (low anterior resection in two, hepatectomy in one), and three using PRRT. Two patients were given palliative chemotherapy and Two with poor performance status were offered best supportive care.

#### Impact of Metastatic Disease on Survival (Fig. 6)

**Fig. 6:**
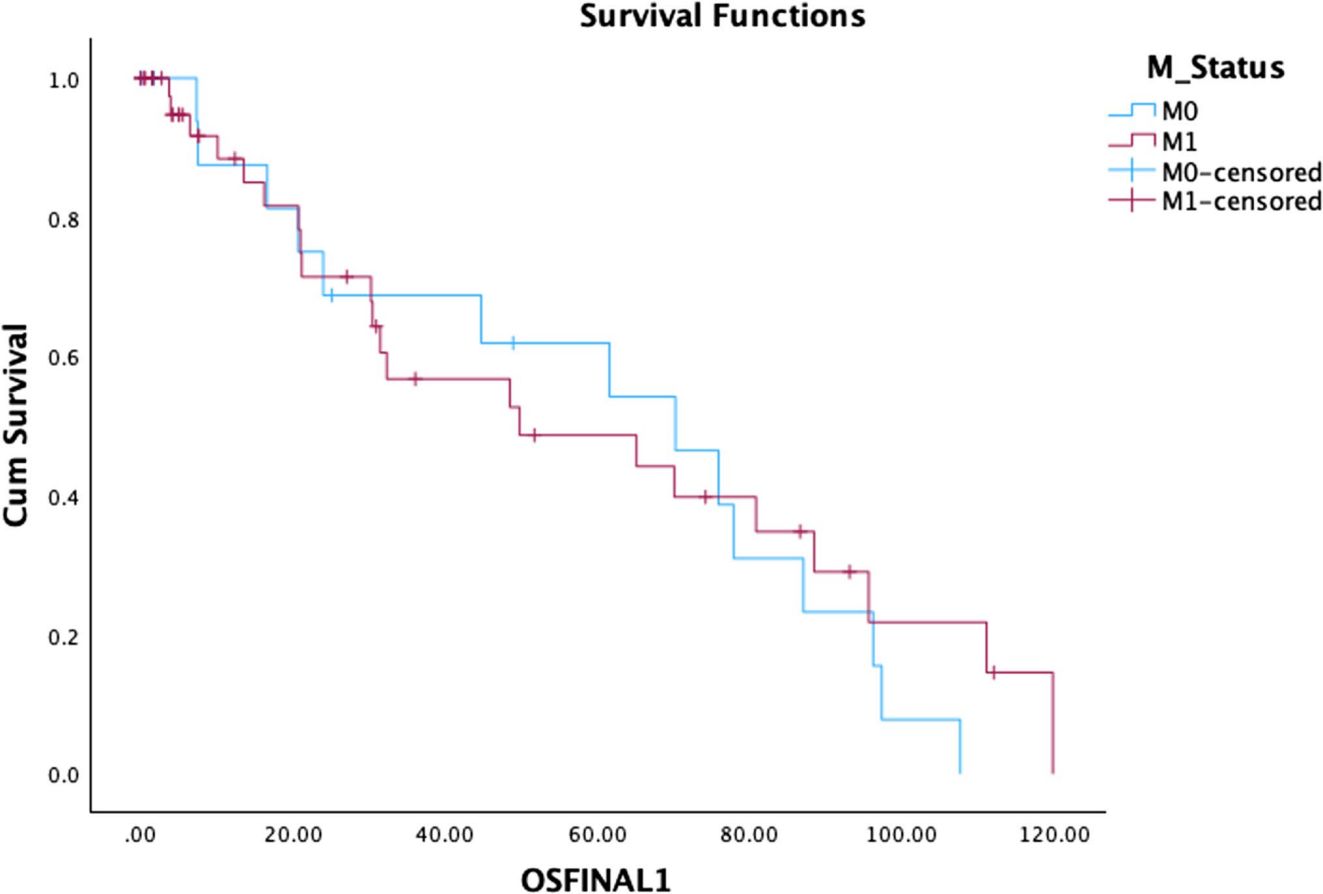
Impact of metastatic disease on OS

In patients with metastatic (M1) disease, the survival was understandably poorer compared to non-metastatic disease (M0). The median survival of M1 disease was 49.78 months (95% CI 0.93–98.6 months), while for M0 disease, it was 70.24 months (95% CI 35.4–105.0 months).

## Discussion

The current study provides valuable insights into the epidemiology, diagnosis, and management of rectal NETs in India. To the best of our knowledge, this is the first study evaluating this disease subset in this geography.

Our patients presented at a slightly younger age with a median age of 50 years compared to the global median of 56–57 years. We also had a slight male preponderance, and the foci was more often in the low rectum compared to published literature where mid-rectal disease predominates. While these could be related to the small sample size of our cohort or the referral bias, this report identified some other key differences of the disease in our region compared to the world. Patients presented with both clinically advanced disease and a more aggressive tumour biology compared to those observed in other regions. Contrasting with Western data, our patients presented with larger tumour sizes (37 mm) compared with a global median of 10–20 mm, more advanced stages (81% presented with T2 + disease compared with less than 20% globally), and poorer disease grades (82% had Grade II/III disease compared with 80–90% having Grade I globally) [12–14].

For Grade I disease, the lesion sizes observed aligned with global data. However, the predominance of higher-grade lesions likely contributes to the overall larger median tumour size observed. While late presentation, non-specific symptoms, and a lack of screening colonoscopy may explain the larger tumour sizes and advanced AJCC stages, it is crucial to note that many of our patients had higher-grade tumours, elevated MIB/Ki-67 indices, and increased serum CgA levels—all markers of recurrence risk and aggressive tumour biology [15]. This pathological aggressiveness contrasts with findings from studies in China [16], South Korea [17], and Japan [18], suggesting potential biological differences in the disease affecting patients in the Indian subcontinent.

Recent research has also suggested the possible role of insulin resistance, metabolic syndrome, and lipid dysregulation, which can be contributory to development of many cancers including NETs [19]. While these risk factors are common in our cohort, data to differentiate correlative vs causative relationship in Indian patients is not available. Reports also suggest that our population may have the CpG island methylator phenotype [20, 21], which could contribute to the poorer outcomes in our cohort.

Globally, rectal NETs present early and when concern for recurrence is low, organ sparing and quality of life decisions take centre stage. As a result, most guidelines recommend endoscopic management in management of localised rectal NETs [22, 23]. While modified endoscopic mucosal resection (mEMR) is equivalent to endoscopic submucosal dissection (ESD) in < 10 mm lesion size, ESD is preferred for > 10 mm lesions and in the salvage setting.

Unfortunately, while endoscopic and organ sparing treatments are the norm globally, most of our patients needed rectal resections and systemic treatment. Locally advanced disease with larger tumours, frequently invading muscularis propria, necessitated surgical resection in a significant proportion. The higher proportion of distant metastatic disease and nodal disease is also reflected in a greater number of patients needing surgical management of nodal and liver disease and frequent need for neo-adjuvant and adjuvant treatments. The benefit of neo-adjuvant treatment we experienced in a subset of our patients is mirrored by a national cancer database review from Cleveland clinic as well [24]. In their cohort, neo-adjuvant chemotherapy improved survival to 30.9 months in patients large (≥ 2 cm), cT4, poorly differentiated primary, or metastatic disease.

The survival outcomes observed in this study, with an estimated 100% 5-year OS in grade I and II disease, align with global data. The better survival in Grade II disease (value of 25 th centile being 93 months for Grade II compared to 87 months in Grade I) could represent impact of adjuvant treatment on survival. However, the poorer outcomes for grade III tumours, with a median survival of 74 months, emphasizes the aggressive nature of higher-grade lesions and the urgent need for more effective treatment strategies.

Our study has several strengths. First, to the best of the authors’ knowledge, this is the first study that investigated rectal NETs in the region. The comprehensive evaluation of a large cohort of patients with rectal NETs, the detailed characterization of tumour features, and treatment outcomes provides valuable data regarding the disease in the region addressing a huge knowledge gap in this understudied region. Our findings also show that in patients where multi-modality treatment achieves downstaging, acceptable outcomes can be achieved for some patients with aggressive disease, supporting this approach in patients with advanced disease, where 5-year OS of 40% was achievable.

Apart from obvious limitations of a retrospective study, coming from a single tertiary referral centre, a potential referral bias cannot be excluded, affecting generalizability of the study. The field of neuroendocrine pathology has evolved significantly beyond mere grading. In our cohort, AJCC staging system of respective years was used and synchronisation could not be done. This lack of synchronisation, lack of detailed subclassification of the pathology as per current guidelines, and the detailed immunohistochemistry panel was also not available for all patients and while not included in this analysis, is an area of future research.

As the first study looking at a potentially important health problem, our study can not only be a benchmark for the region but also highlight treatment challenges in the region or similar demographics and socioeconomic characteristics. The finding of a high proportion of Grade II/III disease in our cohort raises concerns regarding possibly aggressive biology of rectal NET in our region, and coupled with greater insulin resistance seen in this region, we believe it should be investigated if our population has a greater susceptibility to rectal NETs.

## Conclusion

In conclusion, this study characterizes rectal NETs in our centre in Western India, highlighting their occurrence in younger patients who often present with locally advanced, perhaps biologically aggressive disease. Beyond emphasizing demographic challenges, our findings suggest that multi-modal treatment could improve outcomes when managing locally advanced rectal NETs, with surgery offering improved outcomes in carefully selected patients. Further research into the pathological features of rectal NETs, including potential racial differences, is warranted, as this may have significant global clinical implications.

## Data Availability

The data that support the findings of this study are under governance of the parent institute and can be made available from the corresponding author, AS, upon reasonable request.
